# Effect of the Time of Salivary Contamination during Light Curing on Degree of Conversion and Microhardness of a Restorative Composite Resin

**DOI:** 10.3390/biomimetics3030023

**Published:** 2018-08-22

**Authors:** Rasoul Sahebalam, Alireza Boruziniat, Fahimeh Mohammadzadeh, Abdolrasoul Rangrazi

**Affiliations:** 1 Oral & Maxillofacial Diseases Research Center, Mashhad University of Medical Sciences, P.O. Box 9177948959, Mashhad, Iran; sahebalamr@mums.ac.ir; 2 Dental Research Center, Mashhad University of Medical Sciences, P.O. Box 9177948959, Mashhad, Iran; BorouziniatA@mums.ac.ir; 3 School of Dentistry, Mashhad University of Medical Sciences, P.O. Box 9177948959, Mashhad, Iran; drmzadeh82@gmail.com

**Keywords:** saliva, contamination, composite resin, degree of conversion, microhardness

## Abstract

Saliva contamination is a major clinical problem in restorative procedures. The purpose of this study was to evaluate the effect of the time of salivary contamination during light curing on the degree of conversion and the microhardness of a restorative composite resin. Eight groups of 10 samples for measuring the microhardness and eight groups of 5 samples for evaluating the degree of conversion were prepared. The samples of each group were contaminated with human saliva at a certain time. The first group (T0) was contaminated before light curing. The specimens in groups T2–T30 were contaminated at 2, 5, 10, 15, 20 and 30 s after the start of light curing, respectively. The samples of group T40 were contaminated after light curing. The degree of conversion and the microhardness of the specimens were measured by Fourier transform infrared (FTIR) spectroscopy and Vickers hardness testing techniques, respectively. The results of this study revealed that there were no significant differences between the groups in terms of the degree of conversion of the composite resin. Consistent with the findings for the degree of conversion, significant differences in the microhardness between the groups were not found. In conclusion, from a clinical point of view, the results of our study showed that the time of salivary contamination (before, during or after light curing of composite resin) has no significant effect on the polymerization (degree of conversion) and one of the important mechanical properties of dental composite resins (microhardness).

## 1. Introduction

Composite resins revolutionized modern restorative dentistry in the 1960s and caused improvements in esthetic quality as well as providing good mechanical and physical properties. A main process in the formation of composite resins is polymerization. Basically, this process involves a chemical reaction of monomer molecules to form polymer chains. The clinical success of composite resins directly depends on the degree of conversion after photopolymerization [1]. The degree of conversion is an important parameter in evaluating the polymerization efficacy and is defined as the percentage of reacted aliphatic C=C bonds from the dimethacrylate monomers that are present in their polymeric matrices [2]. There are several direct and indirect methods of measuring the degree of conversion, such as Fourier transform infrared (FTIR) spectroscopy, Raman spectroscopy, electron spin resonance, infrared spectroscopy, dynamic mechanical thermal analysis and attenuated total reflection [3,4].

Inadequate polymerization has several negative effects, including discoloration, decrease in mechanical properties (such as hardness), weakened bond strength between tooth and restoration, increased water sorption and solubility, potential pulpal damage and higher cytotoxicity [5,6,7,8,9]. The mechanical properties of restorative composite resins play an important role in determining the clinical longevity of restorations [10]. In this way, microhardness is one of the most important properties that are correlated with compressive strength, wear resistance, degree of conversion and color stability [11,12,13,14]. It is defined as the material surface resistance to plastic deformation by indentation.

With increased demand and the use of composite resins as an alternative to amalgam fillings, the contamination issue has become an important problem in dentistry [15,16]. Salivary contamination can have adverse effects on the longevity of the restoration and may lead to sensitivity, tooth discoloration and finally, loss of the restoration [17]. Most caries occurs in areas where complete isolation is difficult, such as near or at the gingival margin where saliva contamination is likely to occur [18]; or when the use of a rubber dam is impossible in lengthy clinical treatment, especially in pediatric dentistry [19,20]. Salivary contamination can occur at any time during a restorative procedure. The effect of salivary contamination on the composite resin bond strength has been the subject of several studies. Some of these papers showed that contamination with saliva decreases bond strength [17,21,22,23], while others found no significant decrease in bond strength after salivary contamination [24,25].

To the best of our knowledge, there have been no previous studies evaluating the effect of the time of salivary contamination of composite resins on the degree of conversion and the microhardness. Therefore, the main goal of this study was to investigate the effect of the time of salivary contamination during light curing on the degree of conversion and the microhardness of a composite resin. The null hypothesis was that the time of salivary contamination during light curing has no significant effect on the degree of conversion or the microhardness of composite resins.

## 2. Materials and Methods

### 2.1. Sample Preparation

This study was approved by the Ethics Committee of Mashhad University of Medical Sciences, Mashhad, Iran (ethical code: IR.MUMS.REC.1392.13). Saliva was collected from a single individual aged 23 years in a sterile beaker and applied immediately to the test specimens. The volume of the saliva sample was set to 100 µL for each specimen. 

**Figure 1:**
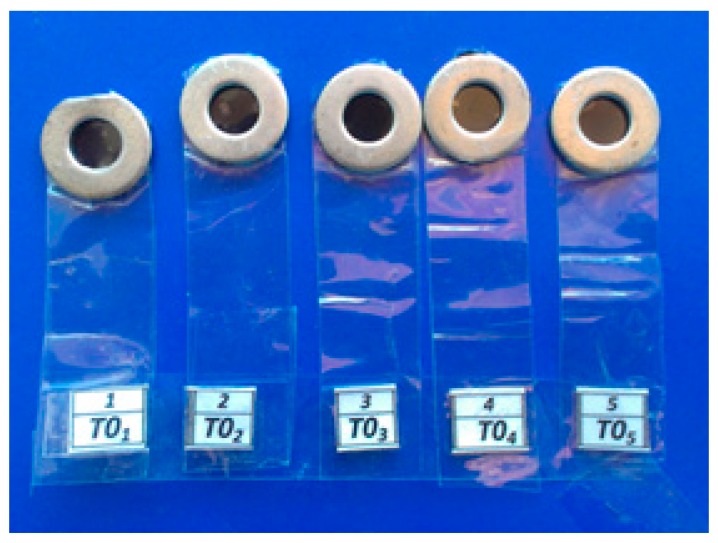
Steel molds used for the preparation of disk-shaped composite resin specimens.

To investigate the effect of the time of salivary contamination during light curing on the microhardness of composite resin, five molds (diameter of 4 mm and thickness of 2 mm, Dental school, Dental Materials Laboratory, Mashhad, Iran) was used to prepare disk-shaped specimens (Figure 1). The mold was filled with a composite resin (Filtek^TM^ Z350 XT, 3M ESPE, St. Paul, MN, USA).

All specimens were light cured for 40 s using a light-emitting diode (LED) curing unit (500 mW/cm^2^, Bluephase^®^ C8, Ivoclar Vivadent AG, Schaan, Liechtenstein). The surface of each specimen was contaminated with human saliva and light curing was performed as follows (eight groups of *n* = 10 samples):

T0: Samples were contaminated with human saliva before the start of light curing, and then light cured for 40 s (negative control).
T2: Samples were contaminated with human saliva 2 s after the start of light curing.
T5: Samples were contaminated with human saliva 5 s after the start of light curing.
T10: Samples were contaminated with human saliva 10 s after the start of light curing.
T15: Samples were contaminated with human saliva 15 s after the start of light curing.
T20: Samples were contaminated with human saliva 20 s after the start of light curing.
T30: Samples were contaminated with human saliva 30 s after the start of light curing.
T40: Samples were light cured for 40 s, before being contaminated with human saliva (positive control).

The samples were stored in distilled water at 37 °C for 24 h before testing. 

### 2.2. Microhardness and Degree of Conversion Measurements

The microhardness of five points was measured with a Vickers hardness tester (Buhler microhardness tester, model no. 1600-6125, Braunschweig, Germany) using a 50 gf load for a 45 s dwell time. The mean of the five indentations’ measurements was recorded as the Vickers hardness. 

To measure the degree of conversion, the specimens were prepared in eight groups of five samples as described above for the microhardness test. The cured specimens were ground into a fine powder by a mortar and pestle, before 2 mg of the powder was mixed with 200 mg of potassium bromide (KBr) powder. Next, KBr pellets were prepared under heavy pressure. The degree of conversion was measured by FTIR spectroscopy (Avatar 370, Thermo Nicolet, San Diego, CA, USA) for both uncured and cured specimens. The degree of the conversion was determined using the changes in the ratios of absorbance intensities of aliphatic C=C (peak at 1638 cm^−1^) to aromatic C–C (peak at 1608 cm^−1^) in the cured and uncured states, according to the following equation:

$$\mathrm{Degree}\mathrm{of}\mathrm{conversion}\;(\%)=(1-\frac{{\left(\frac{A_{aliphatic}}{A_{aromatic}}\right)}_{cured}}{{\left(\frac{A_{aliphatic}}{A_{aromatic}}\right)}_{uncured}})\times 100$$

### 2.3. Statistical Analysis

Statistical analysis was performed using SPSS software (version 11.5, SPSS Inc., Chicago, IL, USA). The data were analyzed by Shapiro–Wilk test to evaluate the normality of the data. Microhardness and degree of conversion data were analyzed by Kruskal–Wallis and one-way analysis of variance (ANOVA) tests, respectively. Statistical significance was set at 5%.

## 3. Results

Descriptive statistics of the microhardness of composite resin for the eight groups are shown in Table 1. None of the microhardness data followed a normal distribution as examined by the Shapiro–Wilk test. The Kruskal–Wallis test (Table 2) demonstrated no significant differences between groups (*p* > 0.05).

The degree of conversion values of the groups is represented in Table 3. The Shapiro–Wilk test confirmed that the values were normally distributed. One-way analysis of variance (ANOVA) showed that the difference in the degree of conversion between the groups was not statistically significant (*p* > 0.05) (Table 4), which followed the same trend as microhardness.

## 4. Discussion

Saliva contamination is a major clinical problem in restorative procedures as it consists of 99% water. Jacobsen et al. [26] indicated that water interferes with the polymerization process of adhesive systems. Similarly, the presence of water may have an adverse effect on the polymerization of composite resins. Therefore, in this in vitro study, the effects of the time of salivary contamination during light curing on the degree of conversion and the microhardness were investigated. Several researchers studied the effects of saliva contamination on bond strength [16,17,19,20,21,22,23,24,25,27,28,29] although the effect of the time of salivary contamination during light curing on the degree of conversion and the microhardness had not been evaluated.

The degree of conversion and the microhardness are two important properties that determine the mechanical and physical behavior of composite resin restorations. In this study, the degree of conversion and the microhardness of the samples were measured with the FTIR and Vickers hardness techniques, respectively, which are some of the most widely used techniques for evaluating the degree of conversion and the microhardness of composite resins. The results of this study revealed that the time of salivary contamination (before, during or after light curing) has no significant effect on the degree of conversion or the microhardness of a restorative composite resin. In other words, it had no significant effect on either the polymerization or mechanical behavior of the composite resin. 

Several studies have shown a good correlation between the degree of conversion and the microhardness of composite resins [30,31,32]. However, some studies did not find this correlation [33,34]. To our knowledge, there have been no studies evaluating the microhardness and the degree of conversion of composite resins, which were contaminated at different times of curing. Most of the studies focused on the effect of salivary contamination on the strength of the composite resin bond to the tooth. Sheikh et al. [35] studied the effect of saliva contamination and cleansing solutions on microtensile bond strengths of self-etch adhesives to dentin. Their results showed that neither saliva nor cleansing solutions adversely affected bond strength. Yoo et al. [23] found that saliva contamination and decontamination methods significantly affected the bond strength of one-step self-etching adhesive systems to dentin regardless of the type of materials evaluated. Kesar et al. [27] evaluated the effect of saliva contamination on the shear bond strength of self- and total-etch adhesive systems on enamel and dentin. Their results revealed that the shear bond strength of a self-etch adhesive system was better than the total-etch adhesive system, while there were no significant differences found within the subgroups of self-etch groups when the adhesive application was done before, after or without saliva contamination. Munaga et al. [22] found that saliva contamination significantly decreased the shear bond strength of the adhesive to dentin. Campoy et al. [36] evaluated the effect of saliva contamination at different stages of the bonding procedure on the bond failure rate of brackets, which were bonded with a hydrophilic self-etching primer. Their results showed that saliva contamination before or after the application of self-etching primer does not increase the clinical risk of bond failure in the bonding of brackets.

Moreover, some studies revealed that using a rubber dam is not necessary to achieve good results. Van Dijken et al. [37] showed that the use of a rubber dam had no effect on the marginal adaptation for anterior composites. Smales et al. [38] found that there were no clinically significant differences in the longevity rates of composite or amalgam, which could be directly related to the use or otherwise of rubber dams. In a review study, Cajazeira et al. [39] summarized that the operatory field isolation technique (rubber dam or cotton rolls) did not influence the longevity of restorations. Brunthaler et al. [40] concluded that the isolation method of the operative field and the professional status of operators (university or general dentist) had no significant effect on composite failure rates. According to these studies, the isolation of the operative field by using a rubber dam has no effect on the longevity of composite restorations. However, saliva contamination may have happened in this situation (without the use of rubber dam). The results of our study demonstrated that saliva contamination during the curing of composite restorations has no adverse effects on the degree of conversion and the microhardness. It should be considered when the light cured composites are polymerized toward the light cure units, the outer surface of composite restoration is first cured. Therefore, this may decrease the water penetration into composite restoration and this can be one of the reasons for the results of the current study, which demonstrated that salivary contamination has no negative effect on the degree of conversion of composite restorations.

Several studies revealed that the bond strength of adhesive systems was decreased by saliva contamination [41,42,43,44]. However, saliva contamination did not have the same effect in different stages of bonding when using an adhesive [41,45]. Our research focused on the effects of the saliva contamination on the step of the application of composite resins. From a clinical point of view, the results of our study showed that the time of salivary contamination (before, during or after light curing of composite resin) has no significant effect on the polymerization (degree of conversion) and one of the important mechanical properties of dental composite resins (microhardness).

## 5. Conclusions

Although previous studies demonstrated that salivary contamination before inserting composite resins may have adverse effects on bonding to tooth structures, the results of our study showed that saliva contamination during light curing of composite restorations has no significant effect on the degree of conversion or the microhardness of these materials.

## Figures and Tables

**Table 1:** Descriptive statistics of the microhardness values of the groups.

Group	Microhardness (VHN)			
	Mean	Standard Deviation	Minimum	Maximum
T0	108.82	15.58	85.50	144.00
T2	103.64	3.74	97.50	110.00
T5	105.62	14.07	89.90	142.00
T10	101.59	6.87	87.90	112.00
T15	109.53	7.05	97.70	120.00
T20	114.74	18.47	101.60	163.30
T30	102.76	5.60	96.20	115.30
T40	107.78	9.61	100.80	132.20

VHN: Vickers hardness number.

**Table 2:** Results of Kruskal–Wallis test for microhardness values between groups.

Group	Mean Rank	*Χ* ^2^	df	*p*-Value
T0	46.55	13.509	7	0.061
T2	34.95			
T5	32.80			
T10	28.40			
T15	53.05			
T20	54.45			
T30	30.65			
T40	43.15			

**Table 3:** Descriptive statistics of the degree of conversion values of the groups.

Group	Degree of Conversion (%)			
	Mean	Standard Deviation	Minimum	Maximum
T0	93.73	2.79	89.25	96.74
T2	96.17	2.10	94.34	98.70
T5	97.49	0.64	96.76	98.36
T10	94.25	2.54	91.06	97.44
T15	94.65	2.79	91.43	97.87
T20	96.83	1.57	95.05	98.81
T30	94.70	3.68	89.68	97.63
T40	95.81	1.69	92.96	97.06

**Table 4:** Results from the one-way analysis of variance (ANOVA) for the degree of conversion.

	Sum of Squares	df	Mean Square	*F*	*p*-Value
Between groups	61.637	7	8.805	1.541	0.189
Within groups	182.852	32	5.714	-	-
Total	244.489	39	-	-	-
